# Head-to-head comparison of [^68^Ga]Ga-DOTA-FAPI-04 and [^18^F]FDG PET/CT for the evaluation of tonsil cancer and lymph node metastases: a single-centre retrospective study

**DOI:** 10.1186/s40644-024-00699-3

**Published:** 2024-05-03

**Authors:** Mengjing Ji, Guang Ma, Cheng Liu, Bingxin Gu, Xinyue Du, Xiaomin Ou, Xiaoping Xu, Shaoli Song, Zhongyi Yang

**Affiliations:** 1 Department of Nuclear Medicine, Fudan University Shanghai Cancer Center, Shanghai, 200032 China; 2 Department of Oncology, Shanghai Medical College, Fudan University, Shanghai, 200032 China; 3 Center for Biomedical Imaging, Fudan University, Shanghai, 200032 China; 4 Shanghai Engineering Research Center of Molecular Imaging Probes, Shanghai, 200032 China; 5 Department of Radiation Oncology, Fudan University Shanghai Cancer Center, Shanghai, 200032 China

**Keywords:** Tonsil cancer, Cervical lymph nodes, Positron emission tomography/computed tomography, [^68^ Ga]Ga-DOTA-FAPI-04, [^18^F]FDG

## Abstract

**Background:**

This study aimed to compare the diagnostic value of [^68^ Ga]Ga-DOTA-FAPI-04 and [^18^F]FDG PET/CT imaging for primary lesions and metastatic lymph nodes in patients with tonsil cancer.

**Method:**

Twenty-one tonsil cancer patients who underwent [^68^ Ga]Ga-DOTA-FAPI-04 and [^18^F]FDG PET/CT scans within two weeks in our centre were retrospectively enrolled. The maximum standardized uptake value (SUVmax) and tumor-to-background ratio (TBR) of the two tracers were compared by using the Mann‒Whitney U test. In addition, the sensitivity, specificity, and accuracy of the two methods for diagnosing metastatic lymph nodes were analysed.

**Results:**

In detecting primary lesions, the efficiency was higher for [^68^ Ga]Ga-DOTA-FAPI-04 PET/CT (20/22) than for [^18^F]FDG PET/CT (9/22). Although [^68^ Ga]Ga-DOTA-FAPI-04 uptake (SUVmax, 5.03 ± 4.06) was lower than [^18^F]FDG uptake (SUVmax, 7.90 ± 4.84, P = 0.006), [^68^ Ga]Ga-DOTA-FAPI-04 improved the distinction between the primary tumor and contralateral normal tonsillar tissue. The TBR was significantly higher for [^68^ Ga]Ga-DOTA-FAPI-04 PET/CT (3.19 ± 2.06) than for [^18^F]FDG PET/CT (1.89 ± 1.80) (p < 0.001). In lymph node analysis, SUVmax and TBR were not significantly different between [^68^ Ga]Ga-DOTA-FAPI-04 and [^18^F]FDG PET/CT (7.67 ± 5.88 vs. 8.36 ± 6.15, P = 0.498 and 5.56 ± 4.02 vs. 4.26 ± 3.16, P = 0.123, respectively). The specificity and accuracy of [^68^ Ga]Ga-DOTA-FAPI-04 PET/CT were higher than those of [^18^F]FDG PET/CT in diagnosing metastatic cervical lymph nodes (all P < 0.05).

**Conclusion:**

The availability of [^68^ Ga]Ga-DOTA-FAPI-04 complements the diagnostic results of [^18^F]FDG by improving the detection rate of primary lesions and the diagnostic accuracy of cervical metastatic lymph nodes in tonsil cancer compared to [^18^F]FDG.

**Supplementary Information:**

The online version contains supplementary material available at 10.1186/s40644-024-00699-3.

## Introduction

With an increasing population and ageing trend, the global cancer burden is increasing. Head and neck squamous cell carcinoma (HNSCC) is the sixth most common cancer worldwide [1]. Squamous cell tonsil carcinoma represents 70–80% of the total of head and neck malignant tumors [2]. There have been remarkable advances in the diagnosis and treatment of tonsil cancer over the past few decades. But patients with tonsil cancer may remain difficult to identify on conventional imaging with CT or MRI [3, 4]. PET/CT has a higher diagnostic value than conventional diagnostic imaging methods (CT and/or MRI) and provides predictive and prognostic insights for head and neck malignancies [5–7]. In addition, the ability to accurately predict prognosis has not progressed accordingly. Accurate staging of cervical lymph nodes is crucial in determining the clinical management and prognosis of patients with tonsil cancer. As with most other solid tumors, prognostic prediction for patients with tonsillar squamous cell carcinoma is largely dependent on tumor-node-metastasis (TNM) staging. But accurate staging has been difficult. Pathological examination is an invasive diagnosis and is often not accepted by patients. Some patients with N0 status suffer from reduced quality of life due to overly invasive diagnostic modalities [8–10]. It has been reported that PET/CT has advantages over CT or MRI alone in the assessment of preoperative staging of HNSCC [11].

The tonsils are a part of Waldeyer's ring and serve as a crucial defense mechanism against inhaled or ingested pathogens by providing initial immune responses [12]. The tonsils are located at the junction of the respiratory and digestive tracts and is constantly exposed to antigens that make it very susceptible to physiological inflammation [13]. Increased physiologic uptake of [^18^F]FDG PET in the oropharynx masks lesions and reduces detection rates. Therefore, tonsil cancer can be challenging to diagnose without invasive procedures due to the limitations of noninvasive imaging techniques like [^18^F]FDG PET/CT. Physiological uptake of [^18^F]FDG in the tonsils can lead to false-positive results, making it difficult to differentiate between normal physiological uptake and pathological uptake indicative of cancer [13–16]. One study stated that the tonsils were the most common site for false positive [^18^F]FDG uptake (39.3% compared to 28.3% for all other sites combined) [17].

Fibroblast activating protein (FAP) is a type II transmembrane glycoprotein that is expressed at low levels in most normal organs and overexpressed in cancer-associated fibroblasts. FAP is involved in a variety of tumor metabolic activities, such as tumor invasion and metastases [18]. FAP is expressed in a variety of tumors [19]. The radiolabelled FAP-targeted inhibitor [^68^ Ga]Ga-DOTA-FAPI-04 has been developed as a novel tracer for PET/CT imaging [20]. [^68^ Ga]Ga-DOTA-FAPI-04 has shown superior diagnostic efficacy over traditional radiotracers in evaluating rare head and neck cancers, including medullary thyroid cancer and tonsil cancer. However, evidence is still limited due to small sample sizes [21, 22]. Therefore, our study included a larger number of patients with tonsil cancer to evaluate the diagnostic value of [^68^ Ga]Ga-DOTA-FAPI-04 and [^18^F]FDG PET/CT imaging for tonsil cancer and metastatic lymph nodes.

## Methods

### Patients

This was a single-centre retrospective study and was approved by the Ethics Committee of our hospital (No.: 2004216–252). All the subjects obtained informed consent to receive [^68^ Ga]Ga-DOTA-FAPI-04 and [^18^F]FDG PET/CT scans and participate in the study. We analysed 21 pathology confirmed tonsil cancer patients who underwent both [^68^ Ga]Ga-DOTA-FAPI-04 and [^18^F]FDG PET/CT within two weeks from September 2020 to September 2022 in our centre. Patients with a history of two or more malignancies were excluded.

### Radiopharmaceuticals

Our centre used the Explora [^18^F]FDG_4_ module with a cyclotron (Siemens CTI RDS Eclips ST) to produce [^18^F]FDG. The [^68^ Ga]Ga-solution was produced by a ^68^ Ga generator (IGG100, Eckert & Ziegler). Radiolabelling of [^68^ Ga]Ga-DOTA-FAPI-04 was performed according to the published procedure [23]. The radiochemical purity of both [^18^F]FDG and [^68^ Ga]Ga-DOTA-FAPI-04 exceeded 95%.

### Imaging and image analysis

Both [^68^ Ga]Ga-DOTA-FAPI-04 and [^18^F]FDG PET/CT scans were performed. For [^68^ Ga]Ga-DOTA-FAPI-04 PET/CT, the activity of the injection was 1.8–2.2 MBq/kg (0.05–0.06 mCi/kg) based on the patient's body weight. For [^18^F]FDG PET/CT imaging, patients fasted for at least 4 h prior to [^18^F]FDG injection (3.7 MBq/kg), and blood glucose levels were below 11 mmol/L. Image acquisition was performed 60 min after injection with a PET/CT scanner (Biograph 16 HR or mCT Flow, Siemens Medical Systems, Knoxville, Tennessee, USA). The spiral CT scan was performed using a standardized protocol (120 kV, 140 mA, slice thickness 3 mm, increments 2 mm, pitch 1.0, rotation time 0.5 s). PET emission scans covering the corresponding regions of the CT were then acquired in 3-dimensional mode using FlowMotion (2–3 min per station) at a rate of 2. Iterative reconstruction of the PET data was performed using ordered subset expectation maximization iterative reconstruction (OSEM) (iterations 2; subsets 21; image size 200). PET is scanned from the patient's brow bone to the upper thigh, followed by a separate acquisition of the cranial area. The duration of the PET/CT scans was 15–20 min.

PET/CT image interpretation was based on visual and semiquantitative analysis and evaluated by two experienced board-certified physicians who did not know the results of the pathology biopsy. We considered that a lesion was suspected on PET/CT when tonsil uptake is greater than contralateral background and lymph nodes are greater than surrounding muscle background. If lesions were found on both sides of the tonsils and focal uptake was higher than the surrounding background, so the situation considered to be positive. The maximum standardized uptake value (SUVmax) and tumor-to-background ratio (TBR) were recorded for primary tumors and neck lymph nodes. The TBR for primary tumors was determined by dividing the SUVmax of the tumor by the SUVmax of the contralateral normal tissue. The TBR for lymph nodes was defined as the SUVmax of the lymph node divided by the SUVmax of the mediastinal blood pool (centre of the aortic arch).

### Diagnostic criteria

The diagnostic criteria included pathological biopsy or clinical follow-up for at least 6 months. The criteria for clinical follow-up were as follows: (1) Tonsillar carcinoma and metastatic lymph nodes—(a) a lesion size that increases significantly without treatment; (b) a lesion size that increases or decreases significantly with antineoplastic therapy; and (c) There was no significant change in the size of the lesion after antitumor therapy, but there was a significant increase or decrease in [^18^F]FDG uptake or enhanced CT/MR had a consistent diagnosis as a malignant lesion during the follow-up period. (2) Benign lesions of the tonsils and lymph nodes—(a) without a substantial change in the size of the lesion untreated; and (b) without a substantial change in the size of the lesion after antitumor therapy but there was a consistent diagnosis as a benign lesion of the tonsils and lymph nodes by enhanced CT/MR during follow-up time. Based on the above diagnostic criteria, PET/CT images were categorized as true positive, false positive, false negative, or true negative. The sensitivity and specificity and accuracy of the PETs for the detection of neck lymph nodes was calculated based on pathology at the time of surgery or based on clinical follow-up for at least six months. If there was at least one lymph node metastasis on PET/CT involving a particular neck side, that neck side was designated to be positive.

### Statistical analysis

All statistical analyses were performed using SPSS 26.0 (IBM). The mean and SD or median and range is used to characterize continuous data. The sensitivity, specificity and accuracy of [^68^ Ga]Ga-DOTA-FAPI-04 and [^18^F]FDG PET/CT for the detection of neck lymph nodes were calculated. The rates of different samples were statistically compared using McNemar's test. Different parameters were compared for independent samples, using the t test for data with a normal distribution and the Mann–Whitney U test for data with a skewed distribution. For correlation analysis between two parameters, Pearson's test was used for normally distributed data, and Spearman's rank correlation coefficient was used for skewed distribution data. *P* values < 0.05 were considered statistically significant.

## Results

### Patient characteristics

A total of 21 patients with squamous cell carcinoma of the tonsils were enrolled in this study, including 5 females and 16 males (mean age, 55.05 ± 8.34 years; range, 38–69 years). The basic patient characteristics are shown in Table 1.

**Table 1:** Characteristics of 21 patients with tonsil carcinoma

Characteristics	N(%)
Gender	
Male	16(76.19)
Female	5(23.81)
Age (years) (mean = 55.05, range, 38–69)	
> 60	7(33.33)
< = 60	14(66.67)
Patients (N = 21)	
Palatine tonsil cancer	13(61.90)
Tongue tonsil cancer	8(38.10)
Histopathologic nature ^a^	
Squamous cell carcinoma	21(100)
HPV (N = 16) ^b^	
Yes	11
No	5
Treatment	
Surgery	13(61.90)
Radiotherapy	19(90.48)
Chemotherapy	18(85.71)
Immunity therapy	2(9.52)
Targeted therapy	1(4.76)
Two or more ^c^	20(95.24)
Neck dissections	
Bilateral	2(9.52)
Unilateral	4(19.05)

^a^Histopathologic nature of primary tumors were squamous carcinoma of the neck

^b^Sixteen of the 21 patients enrolled were tested for HPV

^c^Patients who have undergone multiple treatment regimens

### Diagnostic performance

#### For primary tumors

**Fig. 1:**
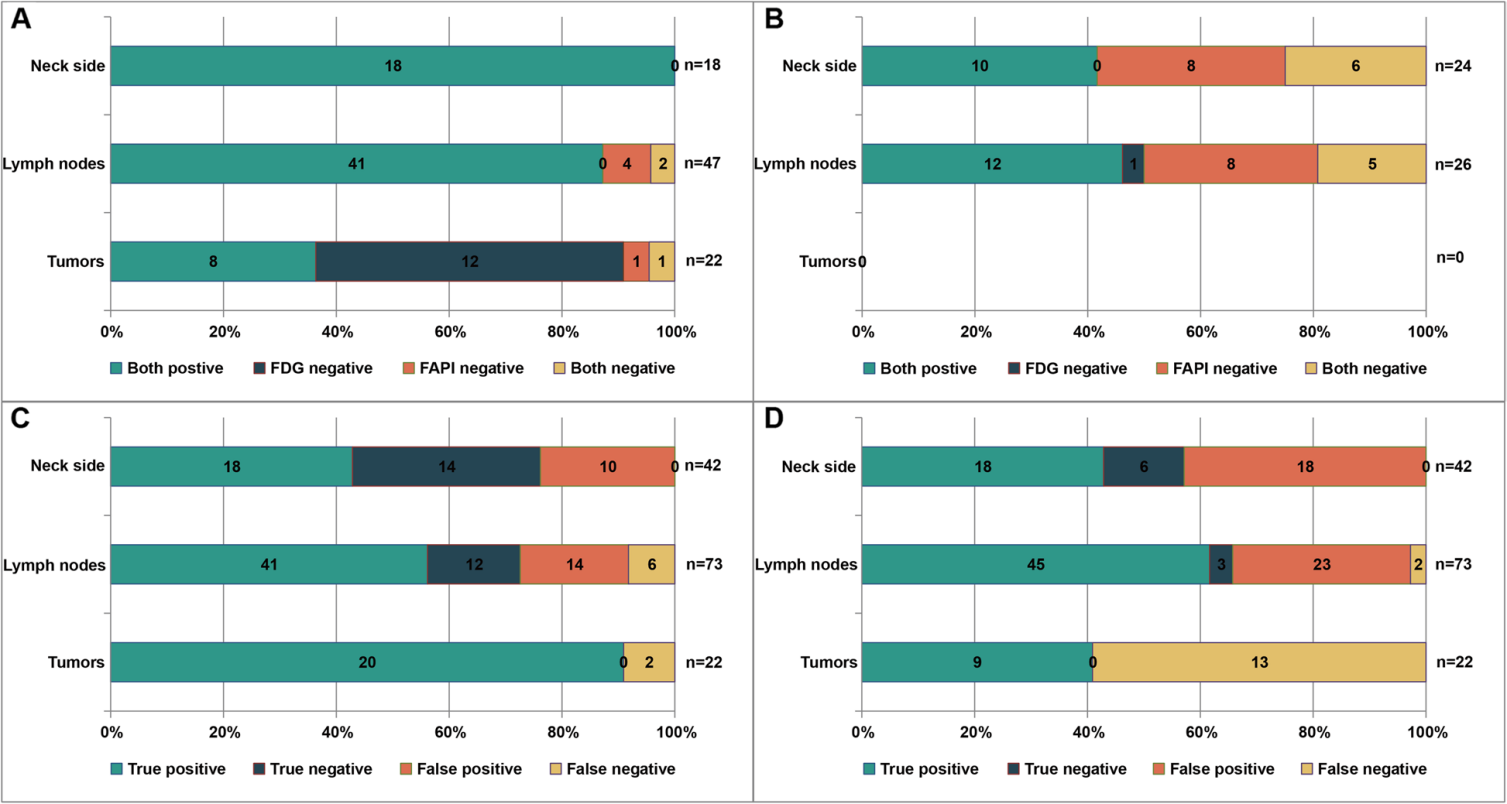
Diagnostic performance of [^68^Ga]Ga-DOTA-FAPI-04 (FAPI) and [^18^F]FDG (FDG) PET/CT in the assessment of tumors and neck region lymph node involvement based on lesions and neck sides. **A** Comparison of [^68^Ga]Ga-DOTA-FAPI-04 and [^18^F]FDG uptake in all positive lesion^a^. **B** Comparison of [^68^Ga]Ga-DOTA-FAPI-04 and [^18^F]FDG uptake in all negative lesion^a^. **C** [^68^Ga]Ga-DOTA-FAPI-04 uptake in all lesions. **D** [^18^F]FDG uptake in all lesions^a^. The nature of the lesion was confirmed on the basis of pathological interpretations and clinical follow-up

**Fig. 2:**
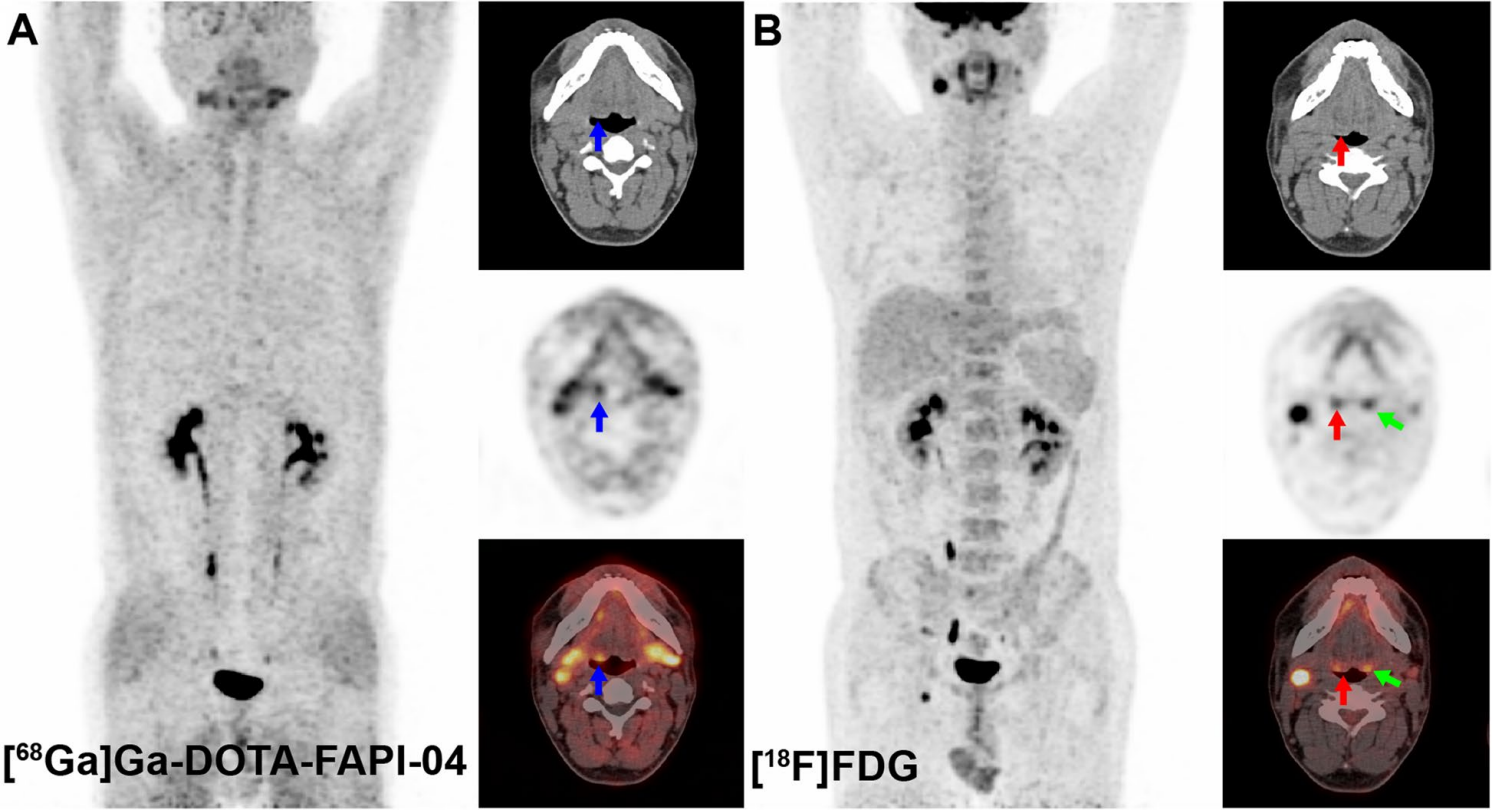
[^68^ Ga]Ga-DOTA-FAPI-04 PET/CT (**A**) and [^18^F]FDG PET/CT (**B**) images of a 39-year-old man with tonsil cancer. [^68^ Ga]Ga-DOTA-FAPI-04 showed high uptake in the primary lesion (blue arrows; SUV 1.53) and no physiological uptake in the contralateral tonsil. [.^18^F]FDG showed lower uptake in the primary lesion (red arrows; SUVmax 3.76) and higher physiological uptake in the contralateral tonsil than in the primary lesion (green arrows; SUVmax 5.76)

Information on each primary tumor is shown in Table 2. A total of twenty-two primary tumors were detected in twenty-one patients by histopathological diagnosis. In one patient, we detected two primary lesions in both sides of the tonsil. The size of the tumor and lymph nodes was measured by contrast-enhanced CT (*N* = 13) or contrast-enhanced MR imaging (*N* = 8). The mean size of the primary tumors was 1.02 ± 0.80 cm, with a minimum and maximum of 0.4 and 4.4 cm, respectively. In the visual analysis, [^68^ Ga]Ga-DOTA-FAPI-04 found 20 primary tumors, while [^18^F]FDG only detected 9 lesions. The sensitivity of [^18^F]FDG PET/CT was 40.91% (9 of 22), compared with 90.91% (20 of 22) for [^68^ Ga]Ga-DOTA-FAPI-04 (*P* = 0.03) (Fig. 1). As we performed a retrospective analysis in which all patients had a positive pathological diagnosis, comparisons of specificity and accuracy could not be made. Representative images of primary tumors on [^68^Ga]Ga-DOTA-FAPI-04 and [^18^F]FDG PET/ CT are shown in Fig. 2.

**Table 2:** Tumor characteristics and imaging results. SUVmax, and TBR of both tracers in the primary tumor and contralateral healthy tonsil

				[^68^ Ga]Ga-DOTA-FAPI-04				[^18^F]FDG			
Patient No	Age	Sex	Size (cm)	Lesion SUVmax	Contralateral normal tissue SUVmax	TBR	Detection^a^	Lesion SUVmax	Contralateral normal tissue SUVmax	TBR	Detection^a^
1	55	M	0.6	2.32	1.42	1.63	TP	4.36	5.03	0.87	FN
2	48	M	0.9	3.13	1.56	2.01	TP	10.33	5.84	1.77	TP
3	58	M	0.9	4.85	1.43	3.39	TP	13.18	2.23	5.91	TP
4	56	M	1.0	2.17	1.48	1.47	TP	1.95	1.50	1.30	FN
5	62	F	1.1	10.63	1.47	7.23	TP	7.72	4.98	1.55	TP
6	65	M	0.4	2.78	1.17	2.38	TP	3.25	2.50	1.30	FN
7	39	M	0.6	3.35	1.53	2.19	TP	3.76	5.76	0.65	FN
8	67	M	0.8	5.00	1.70	2.94	TP	7.33	5.47	1.34	FN
9	66	M	0.8	3.57	1.68	2.13	TP	6.70	5.26	1.27	FN
10	45	F	0.7	3.66	2.72	1.35	FN	6.12	5.58	1.10	FN
11	60	M	1.2	4.04	1.36	2.97	TP	10.77	5.76	1.87	TP
12	58	M	1.0	9.96	1.24	8.03	TP	5.30	5.13	1.03	FN
13	63	M	0.8	7.64	3.22	2.37	TP	6.24	5.99	1.04	FN
14	42	F	1.0	3.11	1.62	1.92	TP	8.96	7.91	1.13	FN
15	52	F	0.9	2.68	2.12	1.26	FN	5.70	3.82	1.49	TP
16	38	F	0.9	5.62	0.99	5.68	TP	10.16	6.18	1.64	TP
17	52	M	1.5	3.62	1.62	2.23	TP	13.47	7.94	1.70	TP
18	64	M	0.6	2.70	1.55	1.74	TP	3.19	3.85	0.83	FN
19	43	M	0.6	2.38	/	/	TP	3.50	/	/	FN
			0.6	2.60	/	/	TP	4.06	/	/	FN
20	69	M	4.4	19.91	3.07	6.49	TP	21.11	2.65	7.97	TP
21	54	M	1.1	4.99	1.15	4.34	TP	16.62	10.24	1.62	TP

^a^*TP* true positive, *TN* true negative, *FP* false positive, *FN*false negative

#### For neck nodal metastasis

**Fig. 3:**
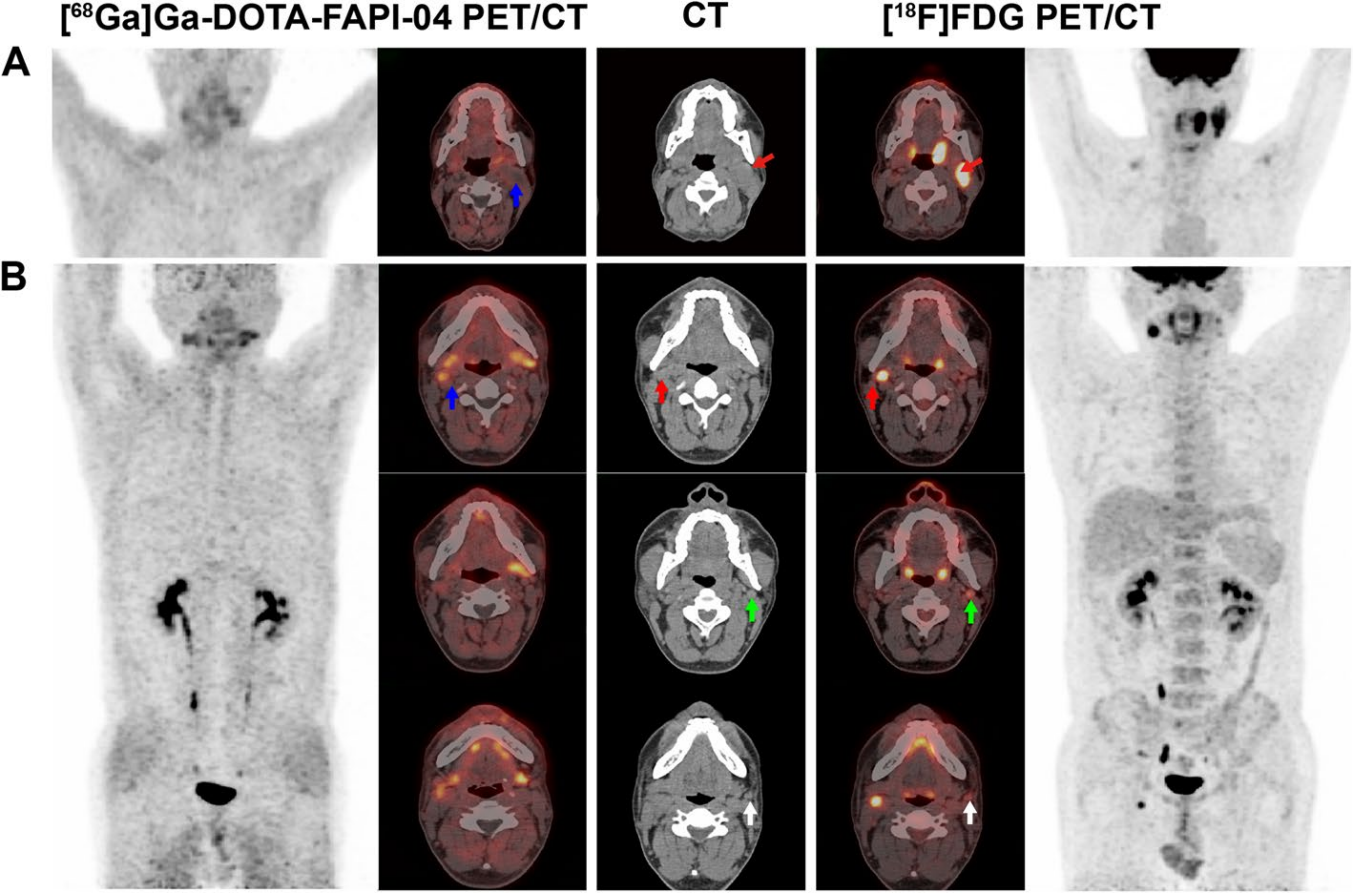
**A** A 60-year-old man with tonsil cancer accompanying metastatic squamous carcinoma of the left neck. CT and [^18^F]FDG PET/CT showed positive lymph nodes on the left side level IIa (red arrow; SUVmax 7.8). [^68^ Ga]Ga-DOTA-FAPI-04 PET/CT showed low uptake of lymph nodes on the left side level IIa (blue arrow; SUVmax 2.5). Metastasis was pathologically confirmed metastasis. **B** A 39-year-old man with tonsil cancer associated with metastatic squamous carcinoma of the right neck. [^18^F]FDG PET/CT detected one lymph node on the right side at level IIa (red arrows; SUVmax 4.8) and two lymph nodes on the left side at level IIa (green arrows; SUVmax 4.2; white arrows; SUVmax 2.9), while [^68^ Ga]Ga-DOTA-FAPI-04 PET/CT detected only one lymph node on the right side at level IIa (blue arrow; SUVmax 12.6). Based on the pathological diagnosis and clinical follow-up, only the right level IIa node was identified as a lymph node metastasis

Information on each lymph node is shown in Supplementary Table S1. A total of 73 lymph nodes were identified by [^68^ Ga]Ga-DOTA-FAPI-04 and [^18^F]FDG PET/CT. According to the pathological interpretations (*n* = 37, 50.68%) and clinical follow-up (*n *= 36, 49.32%), metastatic lymph nodes were found in 17 of 21 patients (80.95%), involving 18 neck sides (42.86%), and 47 lymph nodes (64.38%). Of the 47 lymph nodes, 27 were located in the right level II, 11 in the left level II, 2 in the right level III, 1 in the left level III, 2 in the left level IV and 4 in the left level V. Representative images of lymph nodes on [^68^Ga]Ga-DOTA-FAPI-04 and [^18^F]FDG PET/ CT are shown in Fig. 3.

### Neck side-based analysis

All neck side lesions were detected by [^68^ Ga]Ga-DOTA-FAPI-04 and [^18^F]FDG PET/CT. The representative Table 3 and Fig. 1 display the result. The sensitivity, specificity and accuracy of [^68^ Ga]Ga-DOTA-FAPI-04 and [^18^F]FDG PET/CT for the detection of positive neck lymph nodes in neck side-based analysis were 100.00%(18 of 18) vs. 100.00%(18 of 18), 58.33%(14 of 24) vs. 25.00%(6 of 24), and 76.19%(32 of 42) vs. 57.14%(24 of 42), respectively. The specificity and accuracy of [^68^ Ga]Ga-DOTA-FAPI-04 PET/CT were significantly higher than the corresponding parameters of [^18^F]FDG PET/CT (*P* = 0.008 and *P* = 0.008, respectively) (Table 3). There was no significant difference in the sensitivity of [^68^ Ga]Ga-DOTA-FAPI-04 and [^18^F]FDG (*P* = 1.000) (Table 3).

**Table 3:** Diagnostic performance of [^68^ Ga]Ga-DOTA-FAPI-04 and [^18^F]FDG PET/CT in the assessment of neck region lymph node involvement based on lesions and neck sides

	Sensitivity (%)	Specificity (%)	Accuracy (%)
Based on lesions			
[^68^ Ga]Ga-DOTA-FAPI-04 PET/CT	87.23(41/47)	46.15(12/26)	72.60(53/73)
[^18^F]FDG PET/CT	95.74(45/47)	11.54(3/26)	65.75(48/73)
P	0.125	0.004	< 0.001
Based on neck sides			
[^68^ Ga]Ga-DOTA-FAPI-04 PET/CT	100.00(18/18)	58.33(14/24)	76.19(32/42)
[^18^F]FDG PET/CT	100.00(18/18)	25.00(6/24)	57.14(24/42)
P	1.000	0.008	0.008

### Lesion-based analysis

The representative Table 3 and Fig.1 display the result. The sensitivity, specificity and accuracy of [^68^ Ga]Ga-DOTA-FAPI-04 and [^18^F]FDG PET/CT for the detection of positive neck lymph nodes on lesion-based analysis were 87.23(41 of 47) vs. 95.74%(45 of 47), 46.15%(12 of 26) vs. 11.54%(3 of 26) and 72.60%(53 of 73) vs. 65.75%(48 of 73), respectively. The sensitivity between the two tracers was not statistically significant (*P* = 0.125) (Table 3). The specificity and accuracy of [^68^ Ga]Ga-DOTA-FAPI-04 PET/CT were significantly higher than those of [^18^F]FDG PET/CT (*P* = 0.004 and *P* < 0.001, respectively) (Table 3).

### Comparison of [^68^ Ga]Ga-DOTA-FAPI-04 and [^18^F]FDG Uptake

**Fig. 4:**
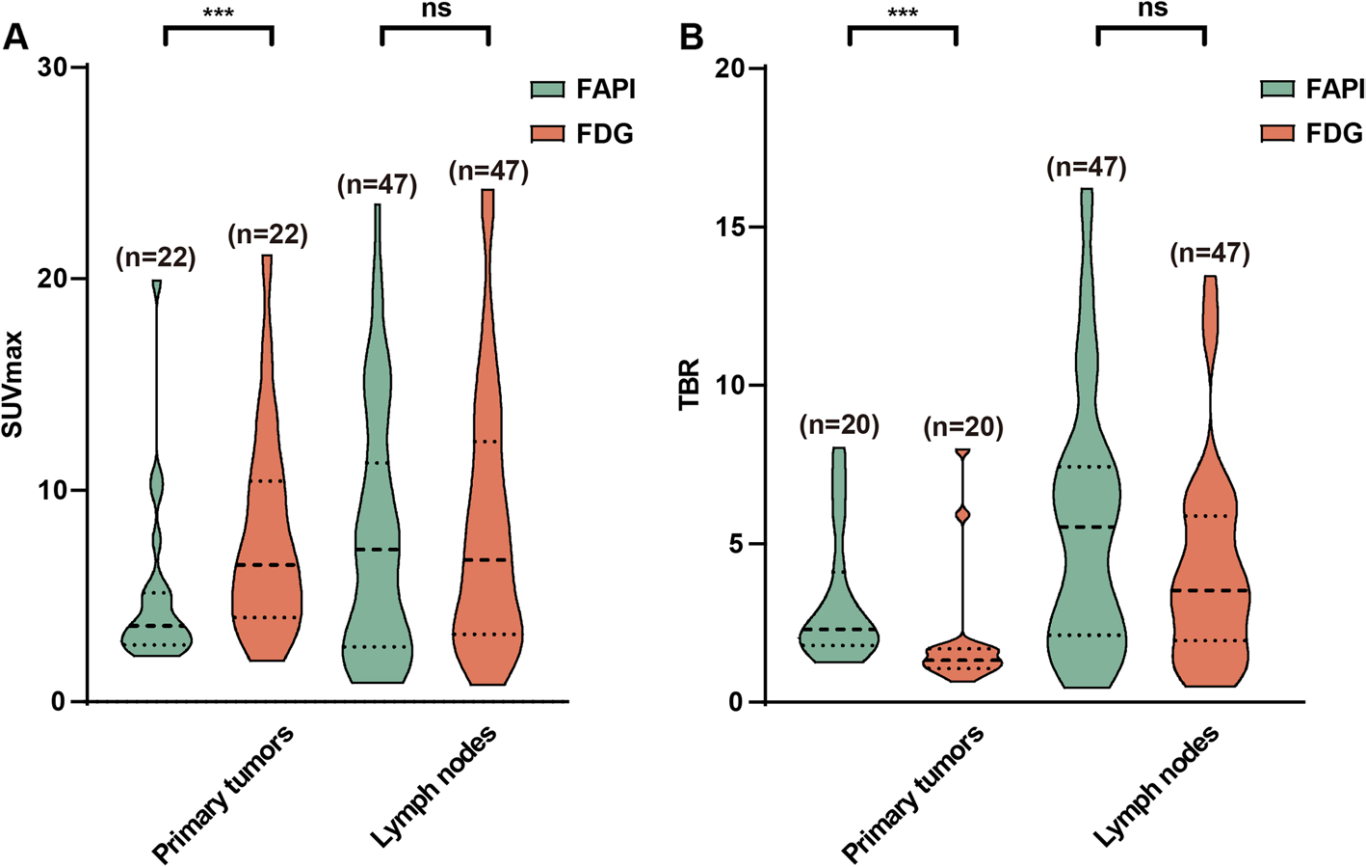
Statistical analysis of [^68^ Ga]Ga-DOTA-FAPI-04 (FAPI) and [^18^F]FDG (FDG) uptake in primary tumors, and neck lymph nodes. **A** The SUVmax of primary tumors and lymph nodes in PET/CT. **B** The TBR of primary tumors and lymph nodes

For all 22 primary tumors, SUVmax was significantly higher for [^18^F]FDG PET/CT than for [^68^ Ga]Ga-DOTA-FAPI-04 PET/CT (SUVmax, 7.90 ± 4.84 vs. 5.03 ± 4.06, *P* = 0.006, Fig. 4). In addition, [^68^ Ga]Ga-DOTA-FAPI-04 PET/CT demonstrated a higher TBR (3.19 ± 2.06) for primary lesions than [^18^F]FDG PET/CT (1.87 ± 1.80) (*p* < 0.001, Fig. 4). The SUVmax and TBR of the primary tumors exhibited a positive correlation with primary tumor size in [^68^ Ga]Ga-DOTA-FAPI-04 (*r* = 0.529, *p* = 0.011 and *r* = 0.456, *p* = 0.043) and [^18^F]FDG PET/CT (r = 0.733, *p* < 0.001; r = 0.671, *p* = 0.001).

[^18^F]FDG and [^68^ Ga]Ga-DOTA-FAPI-04 identified 45 and 41 true positive nodes, respectively. The SUVmax and TBR were not significantly different between [^68^ Ga]Ga-DOTA-FAPI-04 and [^18^F]FDG PET/CT [7.67 ± 5.88 vs. 8.36 ± 6.15 (*P* = 0.498) and 5.56 ± 4.02 vs. 4.26 ± 3.16 (*P* = 0.123), respectively] (Fig. 4). In addition, the SUVmax and TBR of the lymph node showed a positive correlation with lymph node size in [^68^ Ga]Ga-DOTA-FAPI-04 (*r* = 0.621, *P* < 0.001 and *r* = 0.677, *P* < 0.001) and [^18^F]FDG (*r* = 0.681, *P* < 0.001 and *r* = 0.681, *P* < 0.001).

### Changes in TNM staging

Sixteen patients with HPV information were further analysed according to the AJCC VIII (Table 4) [24]. In the assessment of T staging, [^68^ Ga]Ga-DOTA-FAPI-04 diagnosed accurately T-staging in 15 patients while [^18^F]FDG diagnosed 6 patients. As for N staging, [^68^ Ga]Ga-DOTA-FAPI-04 has a similar results with [^18^F]FDG(15 vs. 14). In addition, distant metastases were not identified in 21 patients. Compare to [^18^F]FDG, [^68^ Ga]Ga-DOTA-FAPI-04 identified 9 primary lesions guiding further pathology biopsy and changed clinical management plans.

**Table 4:** Comparison of [^68^ Ga]Ga-DOTA-FAPI-04 (FAPI) and [^18^F]FDG (FDG) PET-based TNM staging in patients with tonsil cancer

Patient No	HPV	Pathological or clinical stage	TNM stage (FDG-based)	TNM stage (FAPI-based)	Staging change (FDG vs. PC^a^)	Staging change (FAPI vs. PC^a^)
1^b^	Negative	T1N2bM0, IVA	N2bM0	T1N2bM0, IVA	NA	None
2	Positive	T1N0M0, I	T1N0M0, I	T1N0M0, I	None	None
3	Negative	T1N2bM0, IVA	T1N2cM0, IVA	T1N2bM0, IVA	N Staging Up	None
6^b^	Negative	T1N2bM0, IVA	N2bM0	T1N2bM0, IVA	NA	None
7	Positive	T1N1M0, I	T0N1M0, I	T1N1M0, I	T staging Down	None
8	Positive	T1N0M0, I	T0N0M0	T1N0M0, I	T staging Down	None
9	Positive	T1N1M0, I	T0N1M0, I	T1N1M0, I	T staging Down	None
11	Positive	T1N1M0, I	T1N1M0, I	T1N1M0, I	None	None
12	Positive	T1N1M0, I	T0N1M0, I	T1N1M0, I	T staging Down	None
13	Positive	T1N1M0, I	T0N1M0, I	T1N1M0, I	T staging Down	None
14	Positive	T1N0M0, I	T0N0M0	T1N0M0, I	T staging Down	None
15	Negative	T1N2aM0, IVA	T1N2aM0, IVA	T0N2aM0, IVA	None	T staging Down
16	Positive	T1N1M0, I	T1N1M0, I	T1N1M0, I	None	None
17	Negative	T1N2bM0, IVA	T1N2aM0, IVA	T1N2aM0, IVA	N staging Down	N staging Down
18	Positive	T1N1M0, I	T0N1M0, I	T1N1M0, I	T staging Down	None
19	Positive	T1N1M0, I	T0N1M0, I	T1N1M0, I	T staging Down	None

^a^Pathological or clinical stage

^b^Patient #1 and Patient #6 were HPV-negative, and no tonsillar carcinoma lesions were detected by [^18^F]FDG PET/CT. T-staging was not available according to the AJCC 8th edition

### HPV-positive and HPV-negative tonsil carcinoma

Sixteen patients underwent HPV testing and eleven patients were diagnosed as HPV positive. For [^68^ Ga]Ga-DOTA-FAPI-04, there were no significant differences in SUVmax (4.43 ± 2.31 vs. 3.25 ± 1.01, *P* = 0.328) and TBR (3.20 ± 2.05 vs. 2.18 ± 0.81, *P* = 0.513) between HPV-positive and HPV-negative patients in the primary sites (Fig. S1). There were also no statistical differences in SUVmax (6.07 ± 4.83 vs. 5.46 ± 4.45, *P* = 0.605) and TBR (4.03 ± 2.96 vs. 4.71 ± 4.03, *P* = 0.817) between HPV-positive and negative patients in the lymph nodes (Fig. S1). For [^18^F]FDG, there were no statistical differences in SUVmax (6.70 ± 2.82 vs. 8.00 ± 4.95, *P* = 0.721) and TBR (1.26 ± 0.40 vs. 2.25 ± 2.07, *P* = 0.371) between HPV-positive and negative patients in the primary sites (Fig. S1). There were also no statistical differences in SUVmax (6.06 ± 3.62 vs. 8.95 ± 8.74, *P* = 1.000) and TBR (3.58 ± 2.63 vs. 4.68 ± 4.55, *P* = 0.957) between HPV-positive and negative patients in the lymph nodes (Fig. S1).

## Discussion

PET/CT has been widely used in the clinical evaluation of HNSCC, and [^18^F]FDG PET/CT has shown substantial diagnostic value in the initial staging and recurrence diagnosis of HNSCC [25]. Several studies reported that FAPI have shown good diagnostic efficacy, especially in the early stage of HNSCC [26–29]. But [^68^ Ga]Ga-DOTA-FAPI-04 in tonsil cancer has not been sufficiently studied to date. Jiang et al. and Promteangtrong et al. comprehensively compared the diagnostic performance of FAPI and FDG in HNSCC, and they concluded that both tracers were comparable in efficiency for primary tumor [26, 27]. However, they were unable to do a detailed subgroup analysis of tonsil cancers. For the neck lymph nodes, our results shown that FAPI exhibited higher accuracy than FDG, which were consistent with those of Jiang et al. but discordant with Promteangtrong et al. Previously, bilateral tonsillectomy and biopsy of the tongue root and nasopharynx were among the methods for detecting primary tumors [21]. However, these invasive tools may cause some sequelae for the patient. For example, postoperative bleeding with fatal consequences occurs in approximately 5% of patients after tonsillectomy [30]. The emerging [^68^ Ga]Ga-DOTA-FAPI-04 tracer may allow patients with tonsil cancer to avoid diagnostic tonsillectomy, leading to a better outcome for patients of small occult cancers. The study by Serfling et al. is the only one thus far to compare in detail the diagnostic efficacy of [^68^ Ga]Ga-DOTA-FAPI-04 and [^18^F]FDG for tonsil cancer, concluding that [^68^ Ga]Ga-DOTA-FAPI-04 PET/CT is more accurate in detecting tonsil [21]. However, there may be some limitations to their study since it included only eight samples.

Our study further confirmed the low uptake of [^68^ Ga]Ga-DOTA-FAPI-04 in the contralateral healthy tonsils of all patients compared to [^18^F]FDG. [^68^ Ga]Ga-DOTA-FAPI-04 can identify malignant tumors at the tonsils with a better TBR, which is consistent with the study by Serfling et al. [21]. The mean SUVmax of [^68^ Ga]Ga-DOTA-FAPI-04 (5.03) and [^18^F]FDG (12.59) in the primary lesions in this study differed from the results of Serfling et al. (16.06 and 21.26). This may be the result of inconsistencies in the number of patients included and in the size (range, 0.8– 1.7 cm vs. 0.4–4.4 cm; mean: 1.09 ± 0.36 cm vs. 1.02 ± 0.80 cm) and the number of primary tumors; [^18^F]FDG may not only show false-positive lesions due to significant physiological uptake at the tonsils, but also false-negative results due to interference by the underlying physiological uptake. The high physiological uptake in the contralateral normal tissue results in a low TBR on [^18^F]FDG PET/CT, even in cases where the TBR is < 1. This results in many lesions being missed at diagnosis. Therefore, [^68^ Ga]Ga-DOTA-FAPI-04 PET/CT scans can be more effective in making an accurate diagnosis of patients with suspected tonsillar cancer.

Cervical lymph node metastasis is one of the most important prognostic factors in HNSCC and influences disease staging and the subsequent clinical management of patients. Previously, [^18^F]FDG PET/CT can accurately identify lymph node metastases, and Chen et al. described that [^68^ Ga]Ga-DOTA-FAPI-04 PET/CT appears to reduce the false positivity for the detection of neck lymph node metastases compared to [^18^F]FDG [28]. The finding is consistent with our discovery, [^68^ Ga]Ga-DOTA-FAPI-04 PET/CT shown low uptake in inflammatory or other reactive lymph nodes. However, whether [^68^ Ga]Ga-DOTA-FAPI-04 has an advantage in detecting lymph node metastasis is still controversial [28]. In our study, [^68^ Ga]Ga-DOTA-FAPI-04 showed low uptake in inflammatory or other reactive lymph nodes. The comparison of the advantages of [^68^ Ga]Ga-DOTA-FAPI-04 and [^18^F]FDG for the detection of positive cervical lymph nodes is still controversial [21, 28, 31, 32]. Our study concluded that more cervical metastatic lymph nodes were detected by [^18^F]FDG PET/CT (45/47) than by [^68^ Ga]Ga-DOTA-FAPI-04 (41/47). In both lesion-based and cervical-based analyses, the diagnostic specificity and accuracy of [^68^ Ga]Ga-DOTA-FAPI-04 PET/CT for cervical metastatic lymph nodes were significantly higher than those of [^18^F]FDG PET/CT (P < 0.001). We found that the specificity of [^68^ Ga]Ga-DOTA-FAPI-04 and [^18^F]FDG for metastatic lymph nodes in our study was lower than that in previous studies in HNSCC [28, 33, 34]. This may be due to the heterogeneity of the population across regions, and the sample may not correctly reflect the characteristics of the population. Additionally, our study only enrolled tonsil cancer patients while other investigations showed more heterogeneity, including HNSCCs other than tonsil cancer, such as laryngeal cancer. We believe that other tools are still needed for the clinical diagnosis of metastatic lymph nodes in HNSCC. Notably, due to the limited spatial resolution of PET/CT some lymph nodes of 5 mm or less in size were not detected by either tracer.

[^18^F]FDG uptake is associated with tumor malignancy and can predict treatment outcome and prognosis [35, 36]. FAP expression is also correlated with tumor progression and development [18, 37]. We analysed the correlation of tumor and lymph node size with the SUVmax and TBR of [^68^ Ga]Ga-DOTA-FAPI-04 and [^18^F]FDG, respectively. We concluded that the SUVmax and TBR in [^68^ Ga]Ga-DOTA-FAPI-04 PET/CT and [^18^F]FDG PET/CT were positively correlated with lesion size (P < 0.05). In the future, we can perform research on the correlation of the uptake of [^68^ Ga]Ga-DOTA-FAPI-04 and [^18^F]FDG PET/CT with tumor pathological features in a larger sample to predict the prognosis of patients.

In addition, HPV not only is an important risk factor for the development of tonsillar carcinoma [38], but also affects the staging and prognosis of tonsillar carcinoma [39]. Our study concluded that HPV positivity does not affect the choice of tracer in patients with tonsil cancer or metastatic squamous carcinoma of the head and neck. However, our study sample with HPV was only sixteen, limiting the statistical power of our results. Further studies involving larger populations will be carried out in the future.

There are some limitations in our study. First, this was a single-centre retrospective study. Second, this preliminary study was based on a small sample size. Therefore, the results need to be further evaluated by large, prospective clinical trial. Third, there was a bias in this study because most of the patients with early stages of tonsil cancer were enrolled.

## Conclusion

[^68^ Ga]Ga-DOTA-FAPI-04 improved the detection rate of tonsillar carcinoma and the diagnostic accuracy of metastatic lymph nodes in tonsillar cancer compared with [^18^F]FDG. The availability of [^68^ Ga]Ga-DOTA-FAPI-04 complements the diagnostic results of [^18^F]FDG. Future prospective studies with more samples are needed to study [^68^ Ga]Ga-DOTA-FAPI-04 PET/CT of tonsillar cancer.

We thank the head and neck cancer multidisciplinary team in our center for the great help to our work.

### Supplementary Information

**Supplementary Materials 1. **

## Data Availability

The datasets generated and/or analysed during the current study are not publicly available due to hospital policy, but are available from the corresponding author upon reasonable request.

## Abbreviations

FAPI: Fibroblast activation protein inhibitor
DOTA: 1,4,7,10-Tetraazacyclododecane-N,N',N,N'-tetraacetic acid
[68 Ga]Ga-DOTA-FAPI-04: [68 Ga]Ga-labelled fibroblast activation protein inhibitor
[18F]FDG: Fluorine 18 fluorodeoxyglucose
HNSCC: Head and neck squamous cell carcinoma
CT: Computed tomography
MRI: Magnetic resonance imaging
PET/CT: Positron emission tomography / computed tomography
TNM: Tumor-node-metastasis
FAP: Fibroblast activating protein
SUVmax: Maximum standardized uptake value
TBR: Tumor-to-background ratio
HPV: Human papillomavirus
