# Supplementary material for: Head-to-head comparison of [68Ga]Ga-DOTA-FAPI-04 and [18F]FDG PET/CT for the evaluation of tonsil cancer and lymph node metastases: a single-centre retrospective study
Source: Cancer Imaging. 2024 May 3;24:56. doi: 10.1186/s40644-024-00699-3 (PMC11069139; doi:10.1186/s40644-024-00699-3)

Head-to-head comparison of [^{68}Ga]Ga-DOTA-FAPI-04 and [^{18}F]FDG PET/CT for the evaluation of tonsil cancer and lymph node metastases: a single-centre retrospective study

Supplementary materials

Table S1 Neck lymph node characteristics and imaging results. SUVmax, TBR of both tracers in lymph nodes

Fig. S1 Statistical analysis of [^{68}Ga]Ga-DOTA-FAPI-04 and [^{18}F]FDG uptake in primary tumors, neck lymph nodes(comparison between HPV-positive and HPV-negative patients). A. The SUVmax of primary tumors and lymph nodes in PET/CT. B. The TBR of primary tumors and lymph nodes

Table S1a Neck lymph node characteristics and imaging results. SUVmax, TBR of both tracers in lymph nodes

Patient No.	Age	Sex	Site [a]	Size(cm)	[^{68}Ga]Ga-DOTA-FAPI-04 Lesion SUVmax	[^{68}Ga]Ga-DOTA-FAPI-04 TBR	[^{18}F]FDG Lesion SUVmax	[^{18}F]FDG TBR	Lymph node metastases [b]	Method of Diagnosis
1	55	M	II(L)	1.70	7.40	6.17	8.20	5.13	Y	Biopsy
			II(L)	3.20	6.40	5.33	10.20	6.38	Y	Biopsy
			III(L)	0.80	2.50	2.08	1.60	1.00	N	Follow-up
			IV(L)	0.60	0.90	0.75	1.20	0.75	N	Follow-up
			II(R)	1.30	2.90	2.42	2.40	1.50	N	Follow-up
2	48	M	II(L)	1.00	1.80	1.38	2.60	1.53	N	Follow-up
			II(R)	1.26	0.90	0.69	2.10	1.24	N	Follow-up
3	58	M	II(R)	2.33	9.60	7.38	24.20	13.44	Y	Biopsy
			II(R)	1.90	7.40	5.69	22.80	12.67	Y	Follow-up
			II(L)	1.00	1.60	1.23	5.30	2.94	N	Follow-up
4	56	M	II(R)	1.50	9.40	7.23	4.20	2.80	Y	Biopsy
			II(R)	2.40	8.00	6.15	5.60	3.73	Y	Follow-up
			II(L)	1.20	2.50	1.92	3.00	2.00	N	Follow-up
			I(L)	1.50	2.70	2.08	2.80	1.87	N	Follow-up
5	62	F	II(L)	1.80	4.30	3.07	6.30	3.32	Y	Biopsy
			II(L)	1.70	10.40	7.43	6.70	3.53	Y	Follow-up
			II(L)	1.00	3.20	2.29	2.90	1.53	Y	Follow-up
			II(R)	0.70	1.20	0.86	1.70	0.89	N	Follow-up
6	65	M	II(R)	1.20	4.00	4.00	13.50	6.43	Y	Biopsy
			II(R)	3.60	16.20	16.20	24.10	11.48	Y	Follow-up
			II(R)	0.90	2.20	2.20	2.90	1.38	Y	Follow-up
			III(R)	0.70	2.60	2.60	2.20	1.05	Y	Follow-up
			II(L)	0.70	1.90	1.90	2.00	0.95	N	Follow-up
7	39	M	II(R)	1.70	4.80	4.36	12.60	7.00	Y	Biopsy
			I(L)	0.90	1.80	2.55	4.20	2.33	N	Follow-up
			II(L)	0.90	2.60	2.36	2.90	1.61	N	Follow-up
8	67	M	II(L)	1.10	2.80	1.65	1.80	1.00	N	Follow-up
9	66	M	II(R)	2.40	12.90	8.06	10.10	4.21	Y	Biopsy
			II(R)	0.80	1.30	0.81	3.10	1.29	Y	Follow-up
			III(R)	1.00	1.70	1.06	2.90	1.21	Y	Follow-up
10	45	F	II(L)	0.70	2.20	1.29	2.60	1.08	N	Follow-up
11	60	M	II(L)	2.80	2.50	3.00	7.80	4.59	Y	Biopsy
			II(L)	1.80	2.80	2.80	5.90	3.47	Y	Follow-up
			II(R)	0.90	1.00	1.00	1.90	1.12	N	Follow-up
12	58	M	II(R)	2.40	13.80	6.90	9.40	5.88	Y	Biopsy
			II(R)	1.80	14.90	7.45	7.40	4.63	Y	Biopsy
			II(R)	0.70	0.90	0.45	0.80	0.50	Y	Follow-up

a. L=left; R=right.

b. Y=Yes; N=No.

Table S1b Neck lymph node characteristics and imaging results. SUVmax, TBR of both tracers in lymph nodes

Patient No.	Age	Sex	Site [a]	Size (cm)	[^{68}Ga]Ga-DOTA-FAPI-04 Lesion SUVmax	[^{68}Ga]Ga-DOTA-FAPI-04 TBR	[^{18}F]FDG Lesion SUVmax	[^{18}F]FDG TBR	Lymph node metastases [b]	Method of Diagnosis
12	58	M	II(R)	1.30	7.40	3.70	3.20	2.00	Y	Follow-up
			II(L)	1.20	1.50	0.75	2.60	1.63	N	Follow-up
13	63	M	II(R)	1.20	7.40	5.69	5.90	2.46	Y	Biopsy
			II(R)	2.50	8.20	6.31	7.20	3.00	Y	Follow-up
			II(L)	1.00	1.40	1.08	2.70	1.13	N	Follow-up
14	42	F	II(L)	1.30	1.30	0.76	2.60	1.44	N	Follow-up
			II(R)	0.80	1.40	0.82	1.10	0.61	N	Follow-up
15	52	F	IV(L)	4.80	11.30	8.07	6.30	3.71	Y	Biopsy
			IV(L)	0.80	2.40	1.71	1.90	1.12	Y	Follow-up
16	38	F	II(L)	2.70	14.50	11.15	14.10	11.75	Y	Biopsy
			II(L)	1.00	2.60	2.00	3.40	2.83	Y	Follow-up
			III(L)	1.25	1.80	1.38	3.70	3.08	Y	Follow-up
			II(L)	0.60	1.30	1.00	1.40	1.17	N	Follow-up
			II(R)	1.00	1.30	1.00	1.50	1.25	N	Follow-up
17	52	M	II(R)	3.55	7.20	6.55	9.52	3.97	Y	Biopsy
			II(R)	0.70	1.40	1.27	1.40	0.58	Y	Biopsy
			II(R)	0.90	1.10	1.00	1.80	0.75	Y	Follow-up
			II(R)	0.90	1.30	1.18	2.80	1.17	Y	Follow-up
			II(R)	1.20	1.40	1.27	2.40	1.00	Y	Follow-up
			II(L)	1.30	1.40	1.27	2.50	1.04	N	Follow-up
			II(L)	1.10	2.40	2.18	3.70	1.54	N	Follow-up
18	64	M	II(R)	2.70	4.60	3.29	4.30	2.53	Y	Biopsy
			II(R)	0.80	2.80	2.00	3.30	1.94	Y	Follow-up
			II(L)	1.00	2.30	1.64	2.30	1.35	N	Follow-up
19	43	M	II(L)	2.20	3.80	2.11	4.00	2.00	Y	Biopsy
20	69	M	II(L)	2.60	19.80	13.20	14.60	5.84	Y	Biopsy
			V(L)	1.30	11.00	7.33	16.10	6.44	Y	Biopsy
			V(L)	1.10	19.20	12.80	16.90	6.76	Y	Biopsy
			V(L)	2.90	23.50	15.67	18.60	7.44	Y	Biopsy
			V(L)	2.60	15.80	10.53	14.60	5.84	Y	Biopsy
			II(R)	2.10	15.30	10.20	12.20	4.88	Y	Biopsy
			II(R)	1.40	8.30	5.53	8.30	3.32	Y	Biopsy
			II(R)	1.30	16.30	10.87	10.10	4.04	Y	Follow-up
			I(L)	1.70	2.20	1.47	2.70	1.08	N	Follow-up
21	54	M	II(R)	2.80	5.40	6.00	12.30	6.15	Y	Biopsy
			II(L)	1.00	1.00	1.11	4.30	2.15	N	Follow-up

a. L=left; R=right

b. Y=Yes; N=No.

Fig. S1 Statistical analysis of [^{68}Ga]Ga-DOTA-FAPI-04 (FAPI) and [^{18}F]FDG (FDG) uptake in primary tumors, neck lymph nodes (comparison between HPV-positive and HPV-negative patients). A. The SUVmax of primary tumors and lymph nodes in PET/CT. B. The TBR of primary tumors and lymph nodes

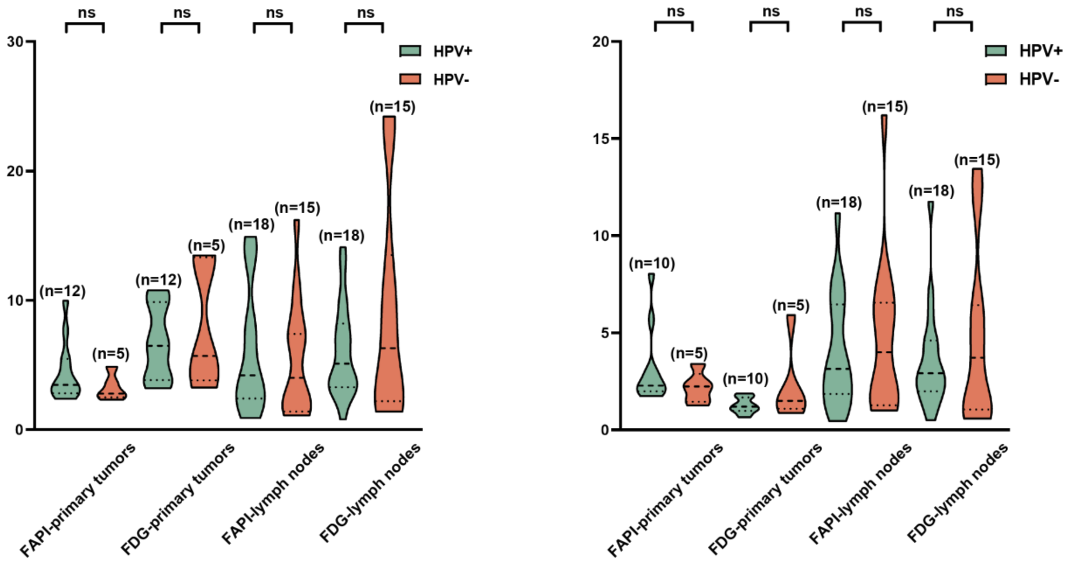

Supplement: Supplementary file 1 — Supplementary Materials 1. [file 40644_2024_699_MOESM1_ESM.pdf]
